# Effects of senktide, a neurokinin 3 receptor agonist, on luteinizing hormone secretion and follicular development in anestrous Shiba goats: a pilot study

**DOI:** 10.1186/1756-0500-7-773

**Published:** 2014-11-03

**Authors:** Natsumi Endo, Tomomi Tanaka

**Affiliations:** Laboratory of Veterinary Reproduction, Tokyo University of Agriculture and Technology, 3-5-8 Saiwai-cho, Fuchu-city, Tokyo, 183-8509 Japan

**Keywords:** Anestrus, Goats, Senktide, LH, Ovulation

## Abstract

**Background:**

Recent studies suggest that neurokinin B and its receptor, neurokinin 3 receptor, have an essential role in the regulation of gonadotropin-releasing hormone and luteinizing hormone (LH) release in several mammalian species. As the first trial, this pilot study reports the effect of intravenous treatment with senktide, a selective agonist of neurokinin 3 receptor, on LH secretion, follicular development in female goats that were clinically diagnosed with anestrus.

**Findings:**

Anestrous goats were intravenously administered 200 nmol senktide at 4-h intervals for 24 h. Most of them examined (5/6 cases) showed a pulsatile increase in LH secretion after each injection of senktide, whereas the remaining one case showed a surge-like increase of LH secretion. Ovulation was confirmed in 5/6 cases at the range of 48–96 h after the beginning of treatment.

**Conclusions:**

This pilot study demonstrated that intravenous treatment with senktide has therapeutic action in goats with anestrus by inducing LH release, which could promote follicular development and ovulation.

## Findings

### Introduction

Neurokinin B (NKB) is a member of the tachykinin family of peptides. Recent studies have revealed that hypothalamic signaling of NKB and its receptor, neurokinin 3 receptor (NK3R), has an important role in the regulation of gonadotropin-releasing hormone (GnRH) neurosecretion in mammalian species [1]. Loss-of-function mutations of *Tac3* and *Tacr3*, which encode NKB and NK3R, respectively, have been identified in patients with hypogonadotropic hypogonadism [2]. Subsequently, research using animal models found that exogenous administration of NKB stimulates neural oscillation governing pulsatile GnRH [3] and luteinizing hormone (LH) secretion [4, 5]. NKB has affinity for other tachykinin receptors, even though marginal, which might compromise the interpretation of the role of NKB/NK3R system in reproductive control [6]. To overcome this problem, senktide was developed as a highly selective and potent agonist of NK3R [7], and currently available only for experimental use. Along with investigating the physiological role of NKB/NK3R system in various stages of the reproductive process, attention is now focused on the therapeutic use of NK3R agonists, e.g. senktide, to accelerate or improve gonadal activity in humans as well as domestic animals [8].

Anestrus is a major component of postpartum infertility in domestic animals including dairy cows [9], which extends calving interval and leads great economic loss for farmers. Particularly in high-yielding dairy cows, occurrence of anestrus is often associated with nutritional deficiency in early lactation period. The condition of anestrus is characterized by the lack of ovarian cycles or gonadal activity with repeated turnover of follicles without ovulation. The existing follicles are incapable of producing sufficient estradiol-17β (E_2_) to induce ovulation, owing to the decreased secretion of GnRH and LH. Taking the endocrine background of anestrus into consideration, anestrous animals are relevant subjects for investigating the therapeutic action of NKB on ovarian dysfunction through the stimulation of GnRH/LH secretion.

Shiba goats are non-seasonal breeders under natural daylight conditions in Japan, and are used as experimental and clinical models for the investigation of reproductive functions in domestic ruminants. Anestrus is also observed in this species, although the incidence is very low under normal management conditions. In this pilot study, we examined the effect of intravenous treatment with senktide on LH secretion and follicular development in anestrous female goats.

### Methods

The study protocol was approved by the University Committee for Use and Care of Animals at Tokyo University of Agriculture and Technology (#22-67). Five Shiba goats (2.4 ± 0.5 years of age; 21.8 ± 3.6 kg of body weight) that were clinically diagnosed with anestrus were subjected to the study. The goats and other herd-mates were maintained in an outdoor paddock and fed a maintenance diet based on alfalfa hay cubes twice daily, under group feeding conditions. The five goats had no estrus signs more than 30 days after the last appearance of estrus. After the expected day of estrus (20 or 21 days after ovulation), no functional corpus lutea and only antral follicles were detected by ovarian ultrasonography (7.5 MHz linear probe, HLS-375 M, Honda Electronics Co., Ltd., Aichi, Japan) and the progesterone concentration in plasma was lower than 1 ng/ml. They received senktide on 48.2 ± 16.8 days after the last appearance of estrus.

On the day of the experiment, a catheter was inserted into the jugular vein. The goats were treated seven times with 200 nmol senktide in 5 ml of saline containing 0.25% dimethyl sulfoxide at 4-h intervals, beginning at 1100 h (designated as 0 h). The senktide solution was injected slowly over 5 minutes via the jugular catheter. The dose of senktide was determined according to a previous study [10] to induce pulsatile LH secretion in ovariectomized Shiba goats. Blood samples (1.5 ml) were collected in heparinized tubes through the catheter at 10-min intervals from -2 to 4 h, at 4-h intervals from 4 to 24 h, at 6-h intervals from 24 to 48 h after the beginning of treatment, and thereafter at 24-h intervals up to 120 h. Plasma was separated and assayed for LH, E_2_ and progesterone [11]. Ovarian ultrasonography was performed 2 days before the treatment, just before the treatment (between 0700 and 0800 h on the experiment day) and then daily until 5 days after treatment. All follicles and corpus lutea that were larger than 2 mm in diameter were recorded. All measured values are presented as means ± SD. Differences between means were evaluated by analysis of variance followed by Dunnett’s multiple comparison test or paired *t*-test. Differences with P <0.05 were considered to be significant.

### Results

**Figure 1:**
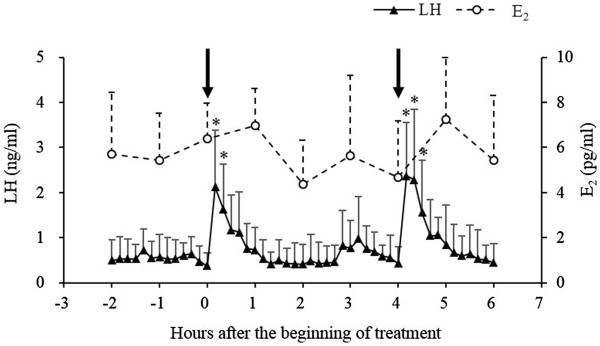
**LH and E**_**2**_ **concentrations from -2 to 6 h after the beginning of senktide treatment.** Arrows indicate the time when 200 nmol senktide was administered intravenously. *: Different significantly (P <0.05) compared with the pretreatment (0 h) value.

Almost all cases (5/6, except for no.9) showed a pulsatile increase in LH concentration in response to each senktide treatment; the LH concentration increased rapidly and reached a peak at 10–20 min after each injection. In these five goats, LH concentrations at 10–20 min after the first injection and at 10–30 min after the second injection were significantly higher (P <0.05) than the pretreatment level (Figure 1). As for E_2_, the mean concentration during the pre-treatment 2 h were 5.9 ± 0.9 pg/ml. The E_2_ concentrations fluctuated and no significant change was observed in the hourly profiles for 6 h after the senktide treatment.

**Figure 2:**
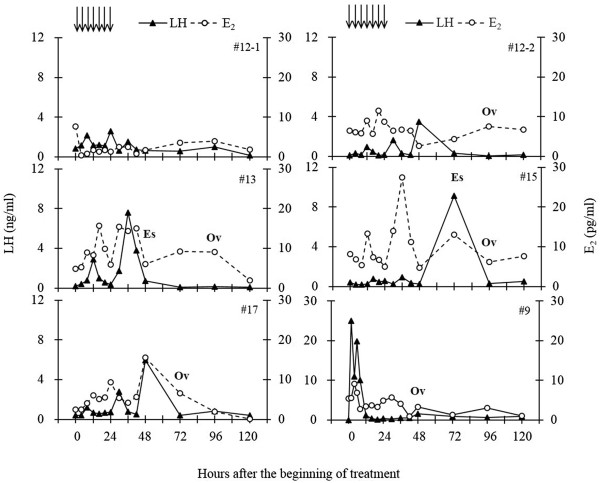
**Profiles of LH and E**_**2**_ **, occurrence of estrus and ovulation in all six goats examined.** Plasma LH and E_2_ concentrations in the samples collected every 4 h from 0 to 24 h, every 6 h from 24 to 48 h, and then every 24 h until 120 h. Arrows indicate the time when 200 nmol senktide was administered intravenously. **Es**: Estrus behavior was detected by visual observation. **Ov**: Ovulation was confirmed by ovarian ultrasonography.

Profiles of LH and E_2_, occurrence of estrus and ovulation in all cases are shown in Figure 2. Estrus and ovulation were observed 2/6 and 5/6 cases, respectively. In the four of five cases that showed a pulsatile increase of LH release, ovulation occurred in response to senktide treatment at 72 (no.17) or 96 h (no.12-2, no.13 and no.15) after the beginning of treatment. Two cases (no.13 and no.15) showed standing estrus one to two days before the day of ovulation. E_2_ level showed a gradual increase through the 24-h repetitive injections. The peak of E_2_ concentration, which was detected at 20–48 h after the beginning of treatment, was preceded or approximately coincided with the peak of LH.

Only one case (no.12-1) failed to ovulate, although a pulsatile increase in LH concentration was clearly observed in response to each injection of senktide. The E_2_ concentration was higher than 5 pg/ml around the time of first injection. However, it decreased to the basal level by 6 h after the beginning of treatment and was maintained lower than 5 pg/ml throughout the experiment.

Goat no.9 showed a different LH response from the other five goats. Mean concentrations of LH and E_2_ during the pre-treatment 2 h in this goat was 4.0 ± 2.5 ng/ml and 8.1 ± 4.7 pg/ml, respectively. A surge-like increase in LH concentration was observed, with a peak of 56.1 ng/ml at 3 h after the first injection. The E_2_ concentration showed a concomitant increase with the increase of LH, followed by a gradual decrease by the time of ovulation (at 48 h after the beginning of treatment).

Goats that ovulated upon the senktide treatment had 2–4 follicles with a diameter larger than 3 mm (mean, 4.0 ± 0.6 mm) on the day of treatment. Finally, 1–4 ovulatory follicles per goat grew after the treatment, and the mean maximal diameter of them was 4.8 ± 0.6 mm. The goat that failed to ovulate (no.12-2) had only one follicle with a smaller diameter of 2.7 mm on the day of treatment, although three additional small follicles were detected 24 h later.

Additional monitoring of ovarian dynamics and blood progesterone concentrations in the ovulated five goats indicated that premature luteal regression occurred (data not shown) and estrus was observed at 5–18 days after the senktide-induced ovulation.

### Discussion

The present study showed the stimulatory action of NKB on the LH secretion in almost all goats with anestrous symptom, in which there was a pulsatile increase in LH concentration in response to each senktide treatment. Recently, it has been revealed that a specific subset of neurons in the arcuate nucleus (ARC) in the hypothalamus, colocalized three neuropeptides, kisspeptin, NKB and dynorphin [12, 13], and is a likely candidate for the intrinsic GnRH pulse generator. Accumulating evidence suggests that the action of NKB or its analog occurs within the ARC through regulation of the release of kisspeptin [3, 4]. An *in vitro* study using cell-attached recording method showed that both NKB and senktide have a potent stimulatory action on the firing of kisspeptin neurons derived from the ARC region of the mouse hypothalamus [14]. In ovariectomized goats, GnRH pulse generator activity at the ARC kisspeptin neurons, which was monitored by recording the multiple-unit activity in the hypothalamus, was stimulated after the intravenous injection of senktide [10]. It is suggested that small-amplitude release of LH (pulsatile LH secretion) is induced by the stimulatory action of NKB on the hypothalamic GnRH pulse generating system.

The present study is the first trial to evaluate the use of NKB for the treatment of anestrus in goats. The obtained findings are limited because there was no untreated control group. In addition, the exact reasons for anestrus remain unclear, but it is likely that insufficient nutrition intake or other related factors such as stress might be attributed to the incidence of anestrus, as has been experimentally demonstrated in feed-restricted animals [15, 16]. Consequently, most cases showed a therapeutic outcome; there was a gradual increase in E_2_ secretion followed by ovulation after the repetitive injections of senktide, at 4-h intervals for 24 h. However, the treatment was insufficient for one goat (no.12) to stimulate follicular development, in spite of the increased LH stimulation caused by senktide treatment. One possible explanation for this is that the failure of follicular development and/or ovulation might be associated with the status of ovarian follicles during senktide treatment. According to ultrasonographic observation of ovaries during the estrous cycle in this species [17], ovulatory follicles appear from 5 days before ovulation and then grow to about 5 to 6 mm in diameter in parallel with the increase in the plasma E_2_ concentration. In addition to the fact that the anovulated goat (no.12) had only one follicle sized 2.7 mm in diameter on the day of treatment, the constantly low level of E_2_ (<5 pg/ml) also indicates the absence of steroidogenically active follicle(s) that can respond to LH stimulation. For optimal treatment of inactive ovary, the timing, duration and route of treatment seem to require adaptation according to the follicular and endocrine conditions.

In one goat (no.9), a prominent increase in LH concentration close to those observed during the preovulatory LH surge was observed following the first injection of senktide. A similar observation was reported from a study by Billings et al. [5], in which a surge-like increase in LH occurred after the treatment of senktide into the third ventricle in ewes during the follicular phase but not during the luteal phase. Although there may be limits to this pilot study as it is based one case, it seems likely that intravenous injection of senktide in a steroid milieu similar to that present during the follicular phase of the cycle can generate a preovulatory GnRH/LH surge, as a trigger of ovulation.

In conclusion, the present pilot study showed that intravenous treatment with senktide can stimulate LH secretion in anestrous goats, suggesting the potential value of NKB application in the treatment of reproductive disorders. The increased release of LH resulted in ovulation in most cases, except for one goat. This different outcome might be associated with the developmental and steroidogenic status of ovarian follicles during senktide treatment.

## Abbreviations

E_2_: Estradiol-17β
GnRH: Gonadotropin-releasing hormone
LH: Luteinizing hormone
NKB: Neurokinin B
NK3R: Neurokinin 3 receptor.
